# A new twist on superantigen-activated autoimmune disease

**DOI:** 10.1172/JCI187567

**Published:** 2025-01-16

**Authors:** Andrew L. Mason, Doaa Waly, Mohammed S. Osman

**Affiliations:** 1 Division of Gastroenterology, and; 2 Division of Rheumatology, Center of Excellence for Intestinal and Immunology Research, University of Alberta, Edmonton, Alberta, Canada.

## Abstract

Superantigen-induced (Sag-induced) autoimmunity has been proposed as a mechanism for many human disorders, without a clear understanding of the potential triggers. In this issue of the *JCI*, McCarthy and colleagues used the SKG mouse model of rheumatoid arthritis to characterize the role of Sag activity in inflammatory arthritis by profiling arthritogenic naive CD4^+^ T cells. Within the diseased joints, they found a marked enrichment of T cell receptor–variable β (TCR-Vβ) subsets that were reactive to the endogenously encoded mouse mammary tumor virus (MMTV) Sag. Arthritis was improved using reverse transcriptase inhibitors. Moreover, depletion of MMTV Sag-activated TCR-Vβ subsets affected the ability of transferred activated CD4^+^ T cells to induce disease in mice with severe combined immunodeficiency (SCID). Further virological studies should determine whether endogenous or exogenous MMTV is necessary or sufficient to trigger inflammatory arthritis in the SKG model.

## Viral superantigen activity intersects with immunodeficiency and autoimmunity

Patients with rheumatoid arthritis (RA) develop a systemic disease manifested by destructive erosive polyarthritis and associated with pneumonitis, lung fibrosis, and rheumatoid nodules. The cause of RA is unknown, but the disease likely occurs as a result of exaggerated and inadequate immune responses from myeloid cells, such as neutrophils, and lymphocyte responses to unrecognized environmental challenges. Even when accounting for immunomodulatory therapy, patients with RA have an increased risk of viral infection and cancer, suggesting that they may have a relative immune-deficient state associated with heightened immune dysregulation (1).

Genetic studies have pointed toward abnormal T cell activation and related signaling pathways in RA by the identification of key risk loci such as *PTPN22* and *CD40* as well as variants of the tyrosine phosphatase *PTPN2,* a negative regulator of T cell receptor (TCR) signaling. Likewise, the SKG mouse model of RA harbors a W163C mutation in ZAP-70 protein that reduces its binding affinity to the CD3ζ chain and attenuates TCR signal intensity (2). Notably, the SKG model recapitulates many of the clinical features of RA, including similar autoantibody profiles and extra-articular manifestations. The SKG hypomorphic ZAP-70 protein leads to defective thymic selection and the development of arthritogenic CD4^+^ cells with hyporesponsive TCR signaling (3).

**Figure 1:**
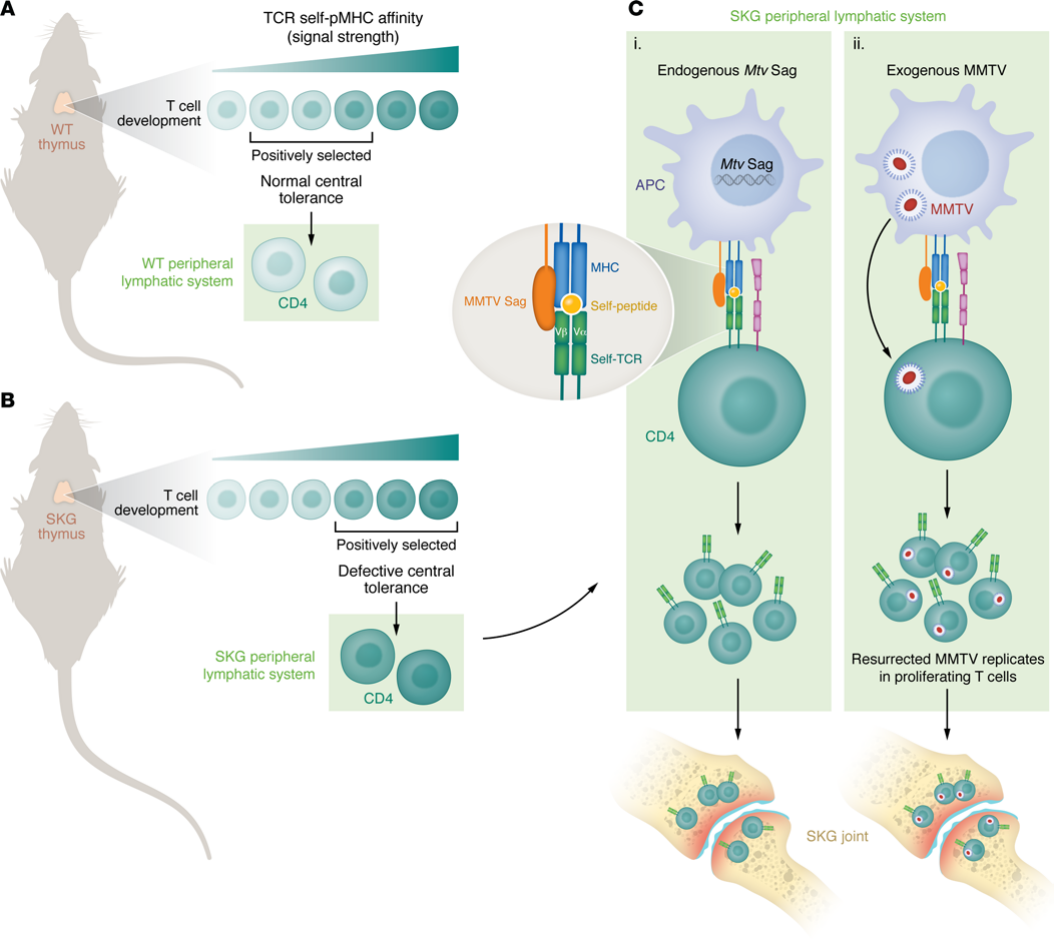
The SKG mouse model of RA supports an endogenous and/or exogenous MMTV Sag-driven autoimmune response. T cells develop in the thymus and progress through positive and negative selection. (**A**) In WT BALB/c mice, thymocytes that react with endogenous MMTV encoded Sags are actively deleted. (**B**) In contrast, the SKG mouse model with a hypomorphic ZAP-70 protein results in positively selected Sag-reactive and autoimmune T cells that have impaired signaling downstream of the TCR/peptide-MHC complex. (**C**) Sags may promote arthritis via two potential models: (i) In the periphery, endogenously encoded *Mtv* Sag activates cognate TCR-Vβ subsets, and these autoimmune naive arthritogenic CD4^+^ T cells home to SKG joints to initiate disease; or (ii) exogenous MMTV arises as a result of resurrected endogenous retroelements in the setting of immunodeficiency and replicates in proliferating cognate TCR-Vβ T cells activated by viral Sags. These lymphocytes home to infect joints, where autoimmune and CD8^+^ cytotoxic T cell responses to MMTV instigate arthritis. pMHC, peptide MHC.

These findings raise the question of how CD4^+^ T cells with a relative impairment of TCR signaling adopt a pathogenic effector state sufficient and necessary to cause arthritis (2, 4). The study reported by McCarthy and colleagues in this issue of the *JCI* addressed the question by profiling naive arthritogenic CD4^+^ T cells in a mouse model that combined the SKG model with a Nur77 TCR signaling reporter (SKGNur) (5). McCarthy et al. identified enrichment of TCR–variable β (TCR-Vβ) subsets recognizing the endogenously encoded mouse mammary tumor virus (MMTV) superantigens (Sags) (5). Microbial Sags can amplify up to 30% of a T cell population by binding the MHC class II of antigen-presenting cells (APCs) with the lateral side of the TCR-Vβ domain (Figure 1). Accordingly, Sag activity is hypothesized to be a potential mechanism for amplification of self-reactive lymphocytes, but the mechanism has not been clarified to any extent in any human autoimmune disease.

In mice, Sag activity is necessary to permit MMTV to replicate in proliferating lymphocytes that passage the virus from the gut-associated lymphoid tissue to the periphery. Relevant to this model, Sag-activated Foxp3^+^ Tregs play a central role in infection by promoting T lymphocyte expansion, while limiting cytotoxic T cell responses to MMTV-infected cells (6). MHC class II polymorphisms also provide a mechanism relevant to the development of autoimmune disease. MMTV Sag efficiently binds the I-E^+^ MHC class II molecule, and as a result, the C57Bl/6 mice lacking I-E^+^ MHC class II are relatively protected from MMTV infection; correspondingly, SKG C57Bl/6 mice are less susceptible to inflammatory arthritis than their SKG BALB/c counterparts.

McCarthy and colleagues used the SKGNur mouse expressing the Nur77/EGFP TCR signaling reporter to identify and isolate antigen-activated naive CD4^+^ T cells prior to the onset of disease. The naive CD4^+^ T cells develop the potential for autoreactivity following adoptive transfer of GFP^hi^ cells into severe combined immunodeficiency (SCID) mice, which then differentiate into arthritogenic IL-17–producing cells (4). Using single-cell RNA-Seq, subsets of the naive CD4^+^ T cells from SKGNur mice shad impaired expression of tolerogenic genes, such as *Socs3,* as well as strongly induced TCR signaling pathway genes consistent with persistent endogenous antigen exposure (5).

Prior studies established that WT BALB/c mice experience a clonal reduction of thymocytes reactive to Sag expressed by endogenous MMTV proviruses (referred to as *Mtv*), whereas SKG mice may encounter the opposite with positive selection of the same T cell subsets as a result of impaired TCR signaling (7). When McCarthy and colleagues performed single-cell TCR analysis of arthritogenic CD4^+^ T cells, they observed enrichment of Vβ3, Vβ5, Vβ7, Vβ11, and Vβ12 subsets within arthritic joints of SKG mice, consistent with cognate Sag engagement from *Mtv-6*, *Mtv-8*, and *Mtv-9* proviruses. Then, in a series of elegant experiments, they established that they could delay the onset of arthritis by treating mice with reverse transcriptase inhibitors (namely tenofovir and emtricitabine) prior to triggering disease in SKG mice with zymosan. Depletion of Sag-reactive subsets in transfer studies of CD4^+^ T cells into SCID recipients also delayed arthritis onset (5).

## Exogenous versus endogenous MMTV Sags

McCarthy and colleagues proposed a model in which impaired TCR signaling in the SKG mouse thymus results in the selection of a more self-reactive repertoire CD4^+^ T cells lacking tolerogenic mechanisms (5). When SKG T cells encounter endogenous *Mtv*-encoded Sag in the periphery, they proliferate and differentiate into arthritogenic IL-17–producing autoimmune effector T cells (Figure 1). Notably, parallels with human endogenous retrovirus (HERV) and Sag activity have been reported in juvenile RA (8).

However, virological studies were not performed, and it is unknown whether the Sag activity was mediated by endogenously encoded *Mtv* Sag expression, or by exogenous MMTV. Another point to consider is the effect of reverse transcriptase inhibitors, which block viral RNA polymerization into cDNA during retroviral replication — or retrotransposition with ERV — rather than preventing the process of ERV RNA transcription from DNA (9). Therefore, if exogenous MMTV is implicated in the process, what is its source? Notably, exogenous infection can emerge in the setting of immune deficiency by so-called “resurrection” of endogenous retroelements to form pathogenic and infectious viral particles (10). Most laboratory mice such as BALB/c mice harbor endogenous MMTV proviruses capable of the phenomena.

A review of other mouse models of Sag-related autoimmune diseases lean toward a role for exogenous MMTV replication. For example, the SvEv129 IL-10^–/–^ model of Crohn’s disease (11) develops spontaneous colitis linked with milk-borne MMTV infection. However, the mice do not develop breast cancer, probably because the viral load in weanling pup stomachs is several log-fold lower than the C3H breast cancer–susceptible line (6). Nevertheless, the IL-10^–/–^ mice generate proinflammatory cellular immune responses to MMTV and exhibit MTV-9 Sag activity enrichment of TCR-Vβ5 and TCR-Vβ11 subsets in the colon, consistent with the Sag effect (12). When treated with combination antiretroviral therapy, the SvEv129 IL-10^–/–^ make a virological response to treatment with reduced MMTV levels in the colon. Additionally, they show improved histologic colitis scores, reduced colonic proinflammatory cytokines, and their microbial dysbiosis reverts back toward the microflora observed in WT mice (13).

Other aspects of the IL-10–KO mouse are consistent with exogenous infection. For example, colitis is restricted to strains with documented exogenous MMTV infection, such as the C3H and 129/Sv background. Indeed, SvEv129 IL-10^–/–^ pups can be protected from developing colitis by cross-fostering with dams without MMTV infection (11). Also, breeding SvEv129 IL-10^–/–^ in a germ-free facility protects against colitis because MMTV uses the microflora for entry into small intestine in weanling pups, while also triggering the production of IL-10 to tolerize the host to infection (14). In this regard, the SvEv129 IL-10^–/–^ model highlights the importance of multiple environmental hits potentiating inflammation. In the absence of IL-10, the MMTV Sag-induced lymphocyte proliferation leads to disturbed gut homeostasis with microbial dysbiosis that in turn triggers colitis. The shared requirement for a microbially conventional environment to activate disease parallels with the SKG model, as mice raised in germ-free conditions are resistant to arthritis (3).

Another example of an MMTV model is the autoimmune biliary disease NOD.c3c4 model derived from the nonobese diabetic (NOD) mouse (15, 16). Following removal of the diabetes risk alleles, NOD.c3c4 mice become protected against diabetes but develop cholangitis with the production of antimitochondrial antibodies (AMAs), diagnostic of primary biliary cholangitis (PBC). NOD.c3c4 mice exhibit increased expression of the characterized mitochondrial autoantigens, pyruvate dehydrogenase complex–E2 (PDC-E2) in bile ducts and lymphoid tissues, as well as MMTV proteins in the same tissue distribution (16). The findings in the NOD.c3c4 model parallel observations from patients with PBC, in whom the production of AMA is thought to stem from overexpression of PDC-E2 in bile ducts and perihepatic lymph nodes (17, 18).

Correspondingly, a human betaretrovirus resembling MMTV has been characterized in patients with PBC and localized to lymph nodes with increased PDC-E2 expression (19). Koch’s postulates have been addressed in vitro using homogenized PBC lymph nodes containing human betaretrovirus and pure isolates of MMTV to trigger the autoimmune phenotype with increased PDC-E2 reactivity with biliary epithelium in coculture studies (18–20). In the NOD.c3c4 model, treatment with tenofovir and emtricitabine leads to abrogation of histological cholangitis, decreased serum alkaline phosphatase, as well as a reduction in MMTV RNA levels in the liver, linking disease with active infection (15). While both the colitis and cholangitis models are more consistent with exogenous infection, more definitive studies will be required to specifically target MMTV in both models to assess whether viral replication is necessary or sufficient to produce disease.

## Questions and future direction

The study by McCarthy and colleagues takes an important step forward by demonstrating a direct role of Sag-fueled CD4^+^ T cells in the development of inflammatory arthritis (5).

What remains to be described is whether direct targeting of MMTV can abrogate the Sag effect, to determine whether betaretroviruses trigger arthritis. Do SKG mice without endogenous MMTV develop arthritis, and if so, do off-target effects of antiretrovirals affect the process? A further challenge will be to discern the potential emergence of exogenous MMTV by assessing replicative intermediates and MMTV proteins in SKG mouse arthritic joints.

Understanding how Sag activity is triggered will begin to address some of the complexity of viral pathogenesis in autoimmune disease, whereas future challenges may include understanding how activated lymphocytes home to joints. Further studies can focus on how Sags modulate nonclassical MHC class II antigen presentation to trigger autoimmune responses. Nevertheless, the role of betaretrovirus infection in promoting immunodeficiency, Sag activity, and autoimmunity is an interesting topic for future research (18).

## Version 1 01/16/2025

Electronic publication

## References

[1] Wasko MC (2004). Comorbid conditions in patients with rheumatic diseases: an update. Curr Opin Rheumatol.

[2] Sakaguchi N (2003). Altered thymic T-cell selection due to a mutation of the ZAP-70 gene causes autoimmune arthritis in mice. Nature.

[3] Takeuchi Y (2020). Impaired T cell receptor signaling and development of T cell-mediated autoimmune arthritis. Immunol Rev.

[4] Ashouri JF (2019). Reporters of TCR signaling identify arthritogenic T cells in murine and human autoimmune arthritis. Proc Natl Acad Sci U S A.

[5] McCarthy E (2025). Endogenous antigens shape the transcriptome and TCR repertoire in an autoimmune arthritis model. J Clin Invest.

[6] Acha-Orbea H (2007). Immune response to MMTV infection. Front Biosci.

[7] Papiernik M (1997). T cell deletion induced by chronic infection with mouse mammary tumor virus spares a CD25-positive, IL-10-producing T cell population with infectious capacity. J Immunol.

[8] Sicat J (2005). Expression of human endogenous retrovirus HERV-K18 superantigen is elevated in juvenile rheumatoid arthritis. J Rheumatol.

[9] Montano-Loza AJ (2009). Cyclosporine A inhibits in vitro replication of betaretrovirus associated with primary biliary cirrhosis. Liver Int.

[10] Young GR (2012). Resurrection of endogenous retroviruses in antibody-deficient mice. Nature.

[11] Madsen K (2002). Normal breast milk limits the development of colitis in IL-10-deficient mice. Inflamm Bowel Dis.

[12] Armstrong H (2024). Mouse mammary tumor virus is implicated in severity of colitis and dysbiosis in the IL-10^–/–^ mouse model of inflammatory bowel disease. Microbiome.

[13] Armstrong H (2023). Mouse mammary tumor virus is implicated in severity of colitis and dysbiosis in the IL-10^–/–^ mouse model of inflammatory bowel disease. Microbiome.

[14] Kane M (2011). Successful transmission of a retrovirus depends on the commensal microbiota. Science.

[15] Sharon D (2015). Impact of combination antiretroviral therapy in the NOD.c3c4 mouse model of autoimmune biliary disease. Liver Int.

[16] Zhang G (2011). Mouse mammary tumor virus in anti-mitochondrial antibody producing mouse models. J Hepatol.

[17] Joplin R, Gershwin ME (1997). Ductular expression of autoantigens in primary biliary cirrhosis. Semin Liver Dis.

[18] Syed H (2022). Linking human betaretrovirus with autoimmunity and liver disease in patients with primary biliary cholangitis. Viruses.

[19] Xu L (2003). Does a betaretrovirus infection trigger primary biliary cirrhosis?. Proc Natl Acad Sci U S A.

[20] Goubran M (2022). Isolation of a Human betaretrovirus from patients with primary biliary cholangitis. Viruses.

